# Respiratory contributions to birdsong—evolutionary considerations and open questions

**DOI:** 10.1098/rstb.2023.0431

**Published:** 2025-02-27

**Authors:** Franz Goller

**Affiliations:** ^1^ Institute for Integrative Cell Biology and Physiology, University of Münster, Münster 48149, Germany; ^2^ School of Biological Sciences, University of Utah, Salt Lake City, UT 84112, USA

**Keywords:** birdsong, respiration, rhythm, energetics, feedback control, ventilation

## Abstract

Respiration plays a central role in avian vocal behaviour by providing the airstream that induces vibration of vocal folds. In this role, respiratory movements dictate the coarse temporal pattern of song, while simultaneously fulfilling its vital functions. Whereas these aspects have been investigated in oscines, little information exists in other taxa. Broad taxonomic information is, however, necessary for addressing questions regarding evolutionary specializations of the respiratory system. Acoustic recordings of unstudied taxa suggest that rapid action by respiratory muscles is a basal trait within birds. In addition to controlling the timing of vocalization, respiratory activity also influences acoustic features such as sound amplitude and frequency. The latter is more strongly influenced by respiratory driving pressure in non-vocal learners. Singing, as a highly dynamic respiratory activity presents an opportunity for studying detailed ventilation patterns and thus could give insight into the basic control of airflow in the avian lung–air sac system. Although we have learned many details of how respiratory control is tied into cortical song control, many open questions remain. Control of respiratory pacemaker circuitry by upstream vocal control centres, respiratory input in initiation of vocalization and the use of online feedback from the respiratory system are all incompletely understood.

This article is part of the theme issue ‘The biology of the avian respiratory system’.

## 1. Introduction

In birds, vocalizations play a critical role in diverse behavioural contexts. Many bird species generate specific, highly complex vocal sequences during the reproductive season, which are an important component of territorial behaviour and play a role in the attraction and courtship of potential mates. This vocal behaviour has been the target of natural and sexual selective forces, which have shaped it into one of the most elaborate acoustic behaviours among vertebrates [1].

Most people are aware of iconic birdsongs, often combining aesthetically pleasing melodies of tonal sounds with rapid tempos. However, across the large diversity of birds, we also find songs that, in contrast, appear simple to the human observer. This large diversity provides an opportunity to assess which features of vocal sequences may have perceptual salience for the intended audience, either territorial rivals or potential mates. Selective forces that have shaped temporal and acoustic features of song clearly are complex and perhaps opposing, as this behaviour must be integrated into life-sustaining behavioural and physiological processes. I define song here functionally as vocalizations in the context of reproduction and distinguish it from other vocalizations, termed ‘calls’, which are used in specific behavioural contexts (e.g. alarm, begging, contact).

### (a) Respiratory system and vocal behaviour

The respiratory system plays a critical role in sound production. While this role has been subject to detailed reviews [2–4], I will here use the physiological bases of sound production to discuss the evolution of mechanical and respiratory dynamics across the diverse avian phylogenetic tree and, in doing so, point towards open questions about how the different, occasionally opposing selective forces have shaped the respiratory contributions to vocal behaviour in birds.

As the main respiratory mechanism of birds is discussed in this issue (‘*Biology of the avian respiratory system: development, evolutionary morphology, function, and clinical considerations*’), I here only focus on aspects directly related to phonation. The respiratory tract provides the aerodynamic force that triggers vibrations of the oscillating tissues and therefore plays a critical role in vocal behaviour. The avian vocal organ, the syrinx, is situated within the unpaired interclavicular air sac near the tracheobronchial junction. This placement allows respiratory pressure conditions to act on the vibratory structures and thus facilitate sound generation. In addition, the suprasyringeal respiratory tract (larynx, glottis, mouth, pharynx and beak) is critically involved in modifying the air pressure in the vocal tract and thus shaping the spectral properties of the emitted vocalization [2–4].

### (b) Sound production mechanisms

Depending on the exact location of the syrinx, it can house one or two sound sources, termed either labia or membranes. Additional membranes may be present on the trachea [5,6]. Typically, each syringeal sound source also constitutes a valve, which allows active regulation of the air passage through antagonistic pairs of muscles. The syrinx is therefore an important component of the avian respiratory system, which needs to operate in tune with respiratory mechanics. This synergistic activity involves active control of the valving action with every breath. For example, activation of syringeal muscles in songbirds occurs at the onset of expiration, and this activation may stabilize the labia and prevent their engagement into vibration [3]. This model is supported by the fact that some species wheeze (e.g. zebra finch, *Taeniopygia guttata*; European starling, *Sturnus vulgaris*) or die of asphyxia (e.g. Java sparrow, *Lonchura oryzivora*; finches) when syringeal muscles are inactivated by resection of the tracheosyringeal nerve [6–8].

Syringeal mechanisms of song production have been reviewed in detail recently (e.g. [3,4]) and are discussed in Adam *et al*. [9] in this issue. To generate sound, the sound sources (labia from here on) are set into vibration by an airstream. The aerodynamic drive critically contributes to the vibration regime of the labia [10–12]. Generation of the driving force is therefore a major role of the ventilatory apparatus of birds.

**Figure 1.**
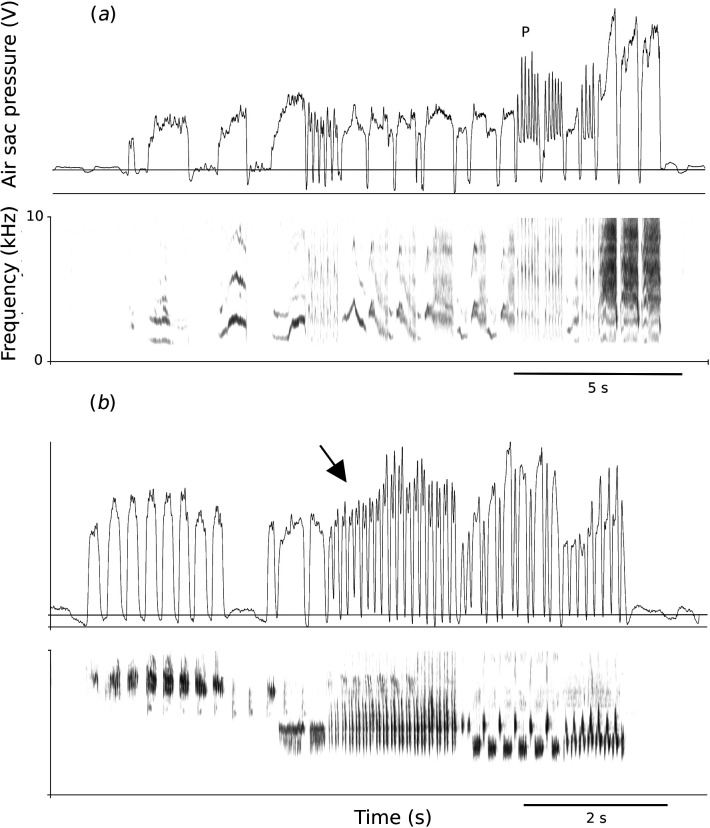
Examples of respiratory patterns (air sac pressure in the anterior thoracic air sac, top panels) generating song (spectrogram, bottom panels) in (*a*) the European starling (*Sturnus vulgaris*) and (*b*) Brewer’s sparrow (*Spizella breweri*). Air sac pressure is displayed as relative output voltage of a Fujikura pressure transducer. The horizontal line indicates ambient pressure. During song, both inspiratory and expiratory pressure are substantially increased relative to quiet respiration (at the beginning and end of each recording). Each pressure pattern shows rapid mini-breath patterns of different duration. A pulsatile syllable is indicated in (*a*) with P; in (*b*) a phrase is started without mini-breaths (arrow) and completed with mini-breaths.

Respiratory dynamics during song have been investigated in fewer than 20 bird species through direct measurements of subsyringeal air sac pressure (typically through cannulation of the anterior or posterior thoracic air sacs). Most vocalizations are generated during expiration (figure 1). Only a few specific sounds are produced during inspiration, including high-frequency elements in the song of zebra finches [13] and the ‘wah’ element after the ‘coo’ in Eurasian collared doves (*Streptopelia decaocto*) [14–16].

**Figure 2.**
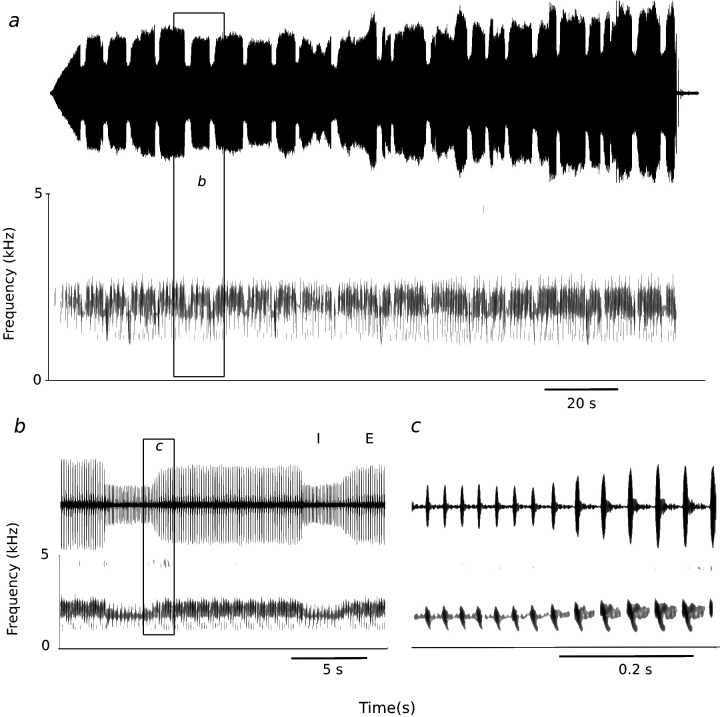
Long songs of the European nightjar consist of trill sequences that are generated while respiration likely switches between expiration (E) and inspiration (I). Assuming this interpretation is correct, respiratory phase switches > 20 times during this song (*a*), and amplitude and frequency characteristics differ for sound pulses generated during presumed expiration (larger amplitude) and inspiration. (*b*)Expansion of the section indicated in (*a*); respiratory phases are indicated by E and I. (*c*) Expandsion of the section indicated in (*b*), showing details of the differences in amplitude and frequency range.

Inspiratory phonation may be more widespread than these few examples—based on physiological data from the small number of investigated species—suggest. For example, the song of the European nightjar (*Caprimulgus europaeus*) is a long trill that can be sustained for minutes. Although air sac pressure has not been measured, sound recordings indicate that the continuous trill is generated over a series of alternating expiratory and inspiratory phases [17], and acoustic features of the trill elements differ between them (figure 2). It is likely that more examples for inspiratory phonation exist across the large diversity of birds.

### (c) The respiratory system and temporal pattern of song

Long song sequences are made possible by short inhalations (mini-breaths) during the silent periods in between song elements (here termed syllables). Mini-breaths typically range in duration from 12 to 80 ms in small birds and are deep, such that the volume of air exhaled during phonation can be replenished [4,13,18–20]. The short mini-breath durations, as confirmed by air sac pressure recordings, are remarkable because the respiratory system of a bird must be reconfigured from an expiratory to an inspiratory position in less than 20 ms. Mechanically, this rapid reconfiguration involves halting the inward trajectory of the ribcage and reversing its movement to generate sub-atmospheric pressure for inhalation. Clearly, both, expiratory and inspiratory muscles must have sufficiently rapid contraction–relaxation kinetics to effect these movements [21–23]. Additionally, trill rates of higher frequencies are generated with sustained expiratory pulses. During these trills (often also called buzzes), expiratory pressure is modulated by activity of the expiratory muscles and sound pulses are generated at the maxima, while silent periods correspond to the minima in pressure. These pulsatile song elements are limited in duration by the available air in the air sac system but produce rapid alternation between sound and silent periods of 50−110 Hz [4]. Although these oscillations generated by the respiratory muscles can be considered rapid, they are greatly surpassed by superfast syringeal muscles, which can modulate airflow up to 250 Hz [24].

The respiratory system therefore contributes the main temporal pattern of song, as it generates the sequence of expiratory pulses corresponding to song elements and intervening silent periods, the mini-breaths, as well as pulsatile song elements. Whereas an alternating pattern of sound and silence is characteristic of most birdsongs, it is particularly impressive in songs containing rapid trills. The potential for generating short mini-breaths facilitates long song sequences with trills whose syllable rate can reach 30 s^-1^ as in the Waterslager canary (*Serinus canaria*), but, in wild species, more typically plateaus near 25 syllables s^-1^ [4,18,25]. Songbird species vary in their composition of song, with some species singing a trill consisting of a single syllable type, while others concatenate different trill sequences (phrases) that feature multiple different tempos. This poses questions regarding to what degree respiratory demands dictate the duration of trills, the sequence of different trill types and song duration.

These questions have not previously been explored in detail. First, song duration does not appear to be limited by demands for oxygen uptake in the few investigated species [26–28]. To the contrary, in some zebra finches and canaries long songs are followed by apneic periods, suggesting that these individuals hyperventilated (i.e. excessive CO_2_ is exhaled) during song and acid/base homeostasis needed to be reestablished [18,27]. Nevertheless, we need data on more species with different temporal patterns of song to fully answer this question.

Second, it is not known to what degree—if at all—respiratory demands dictate sequences of different trill types. In canaries and other species with similar song organization trill rates of the different syllable types can range from a few Hz to > 20 Hz. Even though there are no systematic studies of respiratory demands associated with different syllable types and trill durations, acoustic analyses of canary song suggest that the sequence of trill types is not random [29,30]. It remains to be determined to what degree the observed sequence rules and underlying neural control mechanisms are a consequence of respiratory constraints, such as demand for oxygen, excessive CO_2_ release or limitations on repetitive contraction of expiratory muscles. Alternatively or in addition, perceptual preferences could dictate the sequence of trill types.

### (d) Do rapid mini-breath phrases indicate specialization of respiratory musculature for high speed?

The physiological investigation of a few songbird species documents the temporal dynamics of respiratory movements during song. The highest mini-breath rates (∼ 30 Hz) as well as pulsatile modulation rates (∼ 50−110 Hz) [4] are remarkable in the context of respiratory biomechanics. Expiratory muscles are thin sheets, and the main inspiratory muscles are comparatively small, yet effect respiratory movements of the rib cage and sternum. Are these muscles specialized for driving the fast movements required during rapid trill rates? Data from Bengalese finches (*Lonchura striata*) on contraction kinetics of expiratory muscles show that peak force of individual twitches develops in *ca* 6 ms, which corresponds to the fastest rise times in expiratory air sac pressure [31]. Comparative data on respiratory muscles are not available, such that currently we can only speculate by using comparative data of respiratory rates during different behaviours, including vocalizations, to answer this question.

Normal respiratory rates of birds are lower than those of the other endothermic vertebrate group, mammals [32]. Respiratory patterns underlying echolocation call production in bats do not show a clear 1 : 1 correspondence between respiratory pressure pulse and phonation [33,34]. Sound pulse production can occur during expiration and inspiration, and, at high call rates, multiple calls are generated during one respiratory pulse. Even though bats use high pulse rates (20–30 sound pulses per second), these are not indicative of such high mini-breath rates. Whether bat respiratory muscles can reconfigure the respiratory system as fast as those of birds remains to be investigated.

The vocal behaviour of mice allows a more direct comparison to small oscine songbirds. Vocal sequences of mice are also generated by expiratory pulses separated by inspirations. The shortest expiratory pulses, which generate ultrasound of 26.8 ± 3.6 ms duration, are approximately 40 ms long [35,36]. Importantly, the onset of phonation lagged the initiation of the expiratory pulse by 20.1 ± 3.4 ms, suggesting a less rapid generation in phonatory pressure than is found in small oscines [36]. Fast trills in canaries are composed of syllables of 15 ms duration with intervening silent periods of 15 ms (mini-breaths), thus suggesting more rapid respiratory kinetics.

### (e) Are respiratory movements of song particularly rapid in vocal production learners?

Vocal production learning refers to the ability of imitating sounds that have been acquired from conspecifics or other environmental sources. Only a few bird taxa have been shown to develop songs via such imitation, yet the oscines, parrots and hummingbirds that are capable of vocal learning comprise at least half of all bird species [37–41]. Vocal imitation learning has been linked to more elaborate acoustic and temporal features of song (e.g. [37]), many of which can be attributed to superfast syringeal motor control [4]. Has the evolution of vocal production learning also led to simultaneous specialization of respiratory contributions to song? A comparative study of non-vocal learners and vocal learners should address this question. However, air sac pressure recordings are not available for non-songbirds with rapid syllable sequences. We therefore can only infer respiratory patterns from acoustic recordings. To illustrate this approach, I here compare songs of shorebirds and suboscines, in which song presumably develops innately, with the confirmed mini-breath rates of the vocal production learning oscines.

**Figure 3.**
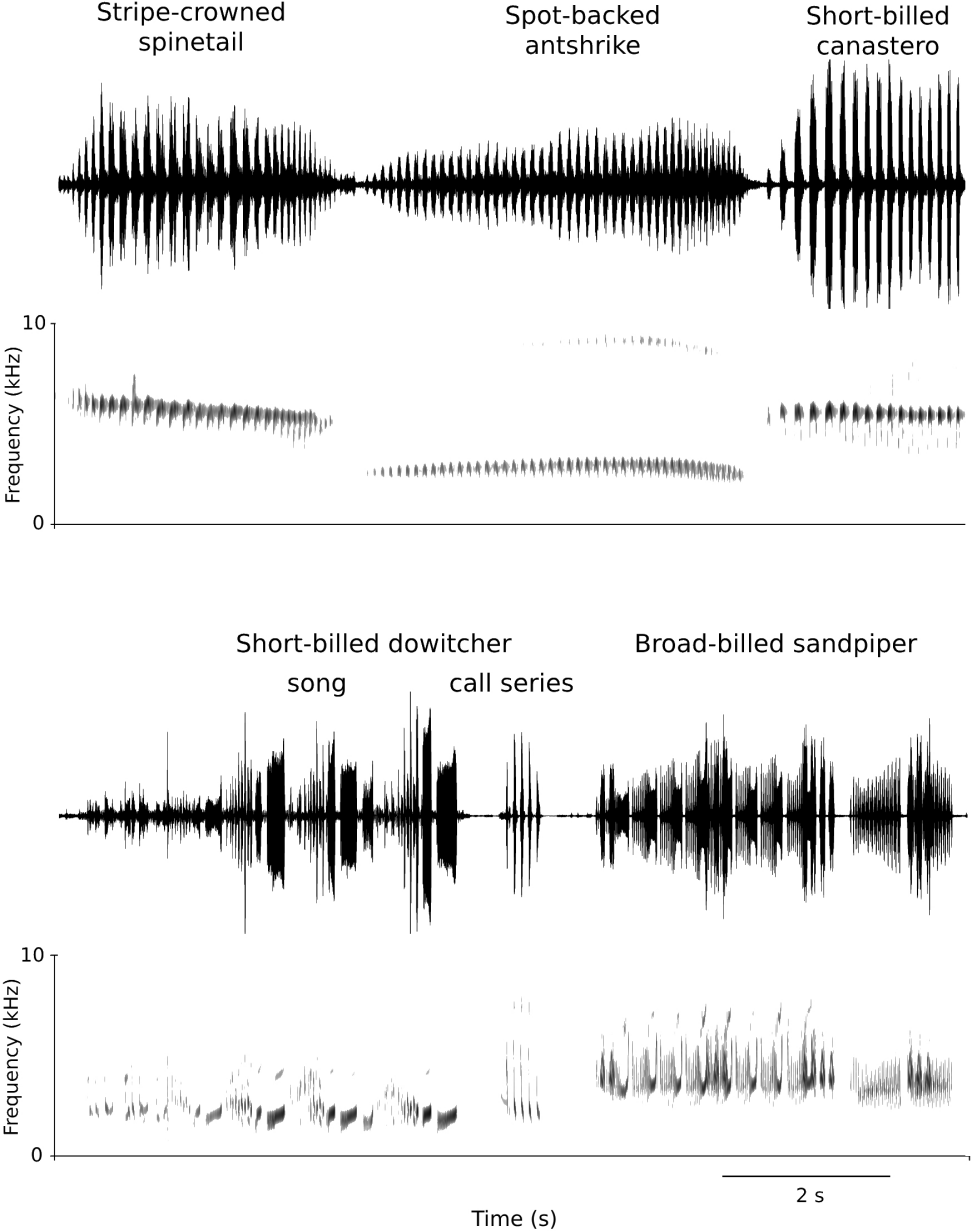
Examples of suboscine (top) and shorebird (bottom) vocalizations (song and call series; displayed as time wave form and spectrogram), which contain short pauses of a duration matching the range of mini-breaths recorded in oscines. The sequences are long, such that replenishing of air is likely necessary. (See table 1 for species details.)

Acoustic analysis suggests that non-vocal learning birds can also display very rapid respiratory patterns. The data from a few small shorebird species show that silent periods can be very short, such that gaps overlap with those found in oscine songs (table 1, figure 3). Because vocal sequences (either song or call series) can be very long, it is likely that the short gaps correspond to mini-breaths. Likewise, some species of the sister taxa to oscines within Passeriformes also display very rapid sound–silence patterns during song (table 1, figure 3). It is therefore likely that very rapid respiratory dynamics have evolved independently of vocal production learning. Nevertheless, the question to what degree selective pressures acting on song have led to rapid kinetics of the respiratory muscles remains unanswered. Comparative physiological studies of species from different bird orders could answer whether specialization for speed has occurred within each taxon .

**Table 1.** Pause duration during song sequences and call series in non-vocal learning suboscines and shorebirds.

species	scientific name	family	pause duration (ms)	sequence duration (s)	XenoCanto source file
suboscines					
Spot-backed antshrike	*Hypoedaleus guttatus*	Thamnophilidae	23−40	~−4.0	XC609535
Stripe-crowned spinetail	*Cranioleuca pyrrhophia*	Furnariidae	28−60	>3.0	XC273238
Short-billed canastero	*Asthenes baeri*	Furnariidae	30	2.9	XC272900
Yellow-billed tit tyrant	*Anairetes flavirostris*	Tyrannidae	7−18	0.9	XC218498
Tufted tit tyrant	*Anairetes parulus*	Tyrannidae	8−30	0.2	XC449803
shorebirds					
Piping plover	*Charadrius melodus*	Calidridae	58−61	>10.0	XC160406
Short-billed dowitcher	*Limnodromus griseus*	Scolopacidae	7−48	~−4.0	XC76194
Dunlin	*Calidris alpina*	Scolopacidae	15−17	2.7	XC833015
Stilt sandpiper	*Calidris himantopus*	Scolopacidae	33−49	3.4	XC203568
Broad-billed sandpiper	*Calidris falcinellus*	Scolopacidae	12−34	~−4.0	XC654231
Western sandpiper	*Calidris mauri*	Scolopacidae	10−70	~−10.0	XC149246
Least sandpiper	*Calidris minutilla*	Scolopacidae	14−86	~−6	XC203720

### (f) Respiration and energetics of song production

As we have seen, respiratory patterns for singing can differ markedly from normal, quiet breathing. Gas exchange is also maintained, such that no significant energetic bottle neck arises even during long song sequences. Additionally, the added movements and underlying neural activity of song cause only a relatively small energetic increase. Singing in canaries, zebra finches and European starlings (*S. vulgaris*) raises oxygen consumption by 1–3% over uptake during pre-song activity [25–27]. Additional evidence that song does not challenge metabolic homeostasis emerged from experiments in which zebra finches sang in a normo-baric but hypoxic atmosphere. Birds still sang readily in oxygen concentrations of 12% and no obvious changes occurred in the temporal pattern of song (F Goller, unpublished observation, 2006).

These measurements from captive birds allow an assessment of the metabolic cost but they do not capture the broad range of behavioural situations in which birds sing in their natural habitats. Many species perform multimodal displays, where song is combined with postural actions and movements, such as dance or flight. These visual displays—especially flight—will raise metabolic rate to much higher levels than observed in the laboratory. Do respiratory constraints arise during these multi-modal displays?

Song production during flight is ideal for addressing such possible respiratory constraints. However, it is obviously very difficult to study respiration during this behaviour physiologically. Again, acoustic recordings can provide a first test. Species with elaborate flight songs have been investigated, such as the skylark (*Alauda arvensis*) [41–45] or tree pipit (*Anthus trivialis*) [46,47]. These data cannot address the question conclusively because skylarks were only recorded during flight song. In tree pipits song structure differs between songs performed during flight and while perched [46]. Flight songs are longer, are composed of more different syllable types and display higher syllable rate but lower phrase rate. These measurements on song structure do not address the question posed here but make clear that the different song structures during flight and while perched make a direct comparison of respiratory patterns less telling. A comparison of durations of the same syllables and associated mini-breaths sung during flight and while perched is needed to assess to what degree flight compromises the respiratory dynamics of singing.

### (g) Air supply and ventilatory patterns during song

The volume of available air in the air sac system is limited and therefore determines how long an expiratory airstream can be sustained. As we have seen, birds are capable of restoring air volume in the air sac system with mini-breaths [13,20]. Additionally, singing more loudly requires higher air sac pressure and presumably more air [48]. It is therefore interesting to ask how much of the available volume of air is used during ‘normal’ singing. We have only few experimental data to address this question. In zebra finches, one or two thoracic air sacs were filled with dental impression medium, and air sac pressure, airflow and sound were compared before and after this reduction in air volume. After injection into one posterior thoracic air sac, air sac pressure and airflow were significantly reduced during song. Additional reduction occurred after the second injection. While pressure amplitude decreased nearly uniformly over the expiratory pulses, the timing of the respiratory pattern remained intact after the first injection. However, after the second injection, air sac pressure declined most drastically toward the end of expiratory pulses and some pulses shortened [49]. These observations indicate that intact zebra finches typically sing with air supply that exceeds the required volume. This safety margin may enable birds to increase the airflow for producing louder songs. To test how much additional air volume is required we need calibrated airflow and air sac pressure measurements (see however the discussion on metabolic cost [48]).

### (h) Direct respiratory control of acoustic parameters

Respiration does not only provide the airstream for phonation but can directly affect acoustic parameters, especially the amplitude and frequency of the vibrations and therefore the emitted sound. The pressure driving phonation directly affects the amplitude of the oscillations of the labia. Additionally, the amplitude can also be controlled by syringeal muscles, which adjust the luminal opening of the syringeal valve and thus position the labia such that maximal flow declination rates vary [10]. For example, an increase in song amplitude was induced in male zebra finches through playback of different background noise levels (Lombard effect). The peak song amplitude increased from 75−89 dB SPL at 50−54 dB noise level to 98−115 dB at 80−86 dB noise level [48]. Calibrated subsyringeal air sac pressure driving song under different noise levels was measured in 5 individuals. An increase in sound amplitude was accompanied by higher air sac pressure (increase from 300−440 mm H_2_O at 50 dB noise to 345−455 mm H_2_O at 75−80 dB noise level; increase of 9–14%). Interestingly, only in one bird did increased air sac pressure result in longer and deeper mini-breaths in between song syllables. The fact that this increased inspiratory activity was not observed in 4 of the 5 individuals indicates that the volume of air expired during high amplitude song may not have been markedly larger, such that a measurable change in inspiratory behaviour could not be detected [48].

Sound frequency is primarily determined by the tension of the labia [10,50,51], and this tension can be modified by the driving air sac pressure and/or by direct action of syringeal muscles. Interestingly, the role of the driving pressure in setting and modulating oscillation frequency appears to be much greater in non-vocal production learning birds than in vocal learners, such as parrots and oscines. Two lines of evidence indicate a drastically different respiratory contribution to frequency control in these two groups. First, there is a strong positive correlation between air sac pressure and fundamental frequency in the few non-learning species that have been studied [47]. Second, denervation of the syringeal musculature has little effect on fundamental frequency in non-learning tyrannids [52], chicks [53], ducks and woodpeckers (F Goller, unpublished, 2016), but it is accompanied by a drastic reduction in fundamental frequency in vocal production learners [10]. Contact calls in the budgerigar show a *ca* 50% drop in frequency [54] and in oscines the drop can be several thousand Hz in some calls (distance call in male zebra finches) and all song elements of oscines, the drop can be several thousand Hz [6,7,55,56]. After bilateral denervation, fundamental frequency in zebra finch vocalizations increases approximately 35 Hz per 100 mm H_2_O (∼ 1 kPa) change in driving air sac pressure [6]. In the great kiskadee (*Pitangus sulphuratus*), a species with innate song development, a doubling in air sac pressure (air sac pressure was not calibrated in this study) yields an increase of approximately a 500 Hz in sound frequency, and this dependence on air sac pressure is the same before and after a bilateral denervation of the syringeal musculature [49]. These results lead to the hypothesis that frequency control via air sac pressure is prominent in non-vocal learning taxa, while control via syringeal musculature is the main regulatory mechanism of vocal learners. Hummingbirds comprise the third avian taxon with many vocal learners and are therefore a group that allows testing of this hypothesis. Additionally, many more non-vocal learning taxa need to be tested.

Another important question arising from these results is how changes in air sac pressure can exert such different effects on the labia. Higher expiratory pressure likely pushes the labia slightly more upstream than lower pressure, thus changing their tension. However, it is not known why this effect is so much greater in oscines and budgerigars than in suboscines and other non-vocal learning groups. A possible explanation may be found in the manner, in which the labia are suspended in the syringeal valve and how syringeal musculature enables or prohibits their movement. Alternatively or additionally, the stress–strain relationship is likely to vary between different labia [56,57], such that muscle activity is necessary to induce strain that is sufficient for an increase in tension in oscines and parrots. This biophysical difference could arise from differences in the protein composition of the extracellular matrix of the labia. Further research is necessary to test these possibilities.

### (i) Respiratory flow patterns during dynamic behaviours

The rapid and intensified respiratory activity during vocal behaviour could affect how the lung is ventilated. The classical model of airflow in the avian respiratory system has been established in anesthetized or resting birds [58]. But is this pattern maintained during song production or other dynamic behaviours? In inactive birds, during inspiration inflowing air is partitioned, with some air perfusing the lung while the remaining volume flows into the posterior set of air sacs. On the following expiration, this air is driven through the lung into the anterior set of air sacs, such that it is exhaled only on the subsequent expiration [58,59]. This model describes the ventilation pattern for most of the air bolus, but some small fraction arrives in the anterior air sacs already at the end of the inspiration, even during respiration at rest (as discussed in [59]). Although experimental data on ventilation patterns during vocal behaviour are scarce, data from call series in yellow-headed blackbirds (*Xanthocephalus xanthocephalus*) suggest that flow patterns might differ during this dynamic behaviour. Tracer pulses of helium injected into the trachea showed different arrival times in the anterior thoracic and interclavicular air sacs, possibly indicating that the behavioural activity generated small pressure differentials. Such differentials have been documented during cooing in doves [60]. Even more interesting is the finding that during short intervals in between calls, some helium was registered in the trachea on the following expiration. This early reappearance of helium in the trachea suggests that some air may bypass the lung during call production, i.e. exit through the mesobronchus [59]. If this interpretation is correct, birds may have some unknown active control over the flow of air in their respiratory system. Considering that breathing patterns during song can be much more rapid and elaborate than during a simple call series, we can expect to find some deviating ventilation patterns. Because it is difficult to trace the airflow pattern during dynamic behaviours, some new technical approach, such as high-speed imaging, may be required to elucidate this question further.

### (j) How is song tied into central neural control of respiration?

The prominent role of respiration in vocal behaviour of birds must be integrated into its main, life-sustaining role. Considering the elaborate and temporally complex respiratory patterns of song, this integration must include motor planning, central coordination and use of feedback, while at the same time maintaining gas exchange and other connected homeostatic control. At the central level, the connectivity between song control centres and respiratory centres has been described, but the actual integration of song into the pacemaker circuitry for breathing has not been elucidated. Similarly, the detailed control mechanisms of the inspiratory and expiratory spinal motor centres are not known.

The respiratory pacemaker circuitry is located in the rostro-caudal column, spanning from pons to the caudal medulla. Inspiratory pre-motor neurons reside in the nucleus parambigualis (PAm) and expiratory pre-motor neurons in the nucleus retroambigualis (RAm) [61–65]. These areas receive input from the dorsomedial nucleus of the intercollicular complex (DM), which is thought to be the nucleus for initiating innate vocalizations. In vocal production learning birds, the cortical premotor circuitry (in oscines HVC, used as a proper name and RA, robust arcopallial nucleus; analogous circuitry in parrots and hummingbirds) projects ipsilaterally to the respiratory centres [66–70].

It can be assumed that meeting respiratory needs is prioritized at all times. Therefore, an upstream signal to vocal sensorimotor and motor control centres in the forebrain is likely such that song initiation can proceed. Such a ‘permissive’ signal has long been proposed, and recent experiments show convincingly that information flow from thalamic regions to approximately 15% of HVC neurons does occur [71]. Its timing suggests that it is related to song initiation. Furthermore, hemispheric connections that facilitate coordination of the two song control circuits in the left and right forebrain exist only at the thalamic and hindbrain levels, suggesting that coordination of the two ipsilateral motor sequences to the syrinx have to originate at these levels. Recordings from HVC neurons projecting to RA in zebra finches show a continuous activation chain, which was interpreted as millisecond-to-millisecond control of vocal motor action. Other models, primarily based on canary song, suggest that motor output from RA may drive a non-linear network of the respiratory pacemaker neurons, such that changes in input frequency or amplitude can drive the various temporal patterns of canary trills [72]. This model is not inconsistent with the data on zebra finch song control. The song in this species is not composed of repeated syllables and may therefore require a more uniform input to the respiratory network. Nevertheless, if the general model holds, a change in activation of the respiratory network is predicted for each of the different syllables of the song. Recordings of this input are currently still missing and a resolution of these different models must await such information. Support for the integrative model comes from experiments in which HVC was cooled with a small Peltier element attached to the skull. In zebra finches, Bengalese finches and canaries initial cooling leads to stretching of the song syllables, but further cooling results in qualitatively altered respiratory patterns, as syllable breaking occurs. Furthermore, inspiration and expiration are not equally stretched during cooling, which might indicate that thalamic input drives in part the overall temporal pattern of song [73–76].

An important aspect of respiratory control during phonation is feedback from the respiratory system. Overall, little is known about the sensory origins of feedback and its online use. The main aspects have been reviewed in detail [3,64,65] and will therefore not be repeated here. New experiments illustrate the potential influence of somatosensory feedback on song motor control [77], but we still lack a detailed understanding of how sensory feedback from different confirmed and putative sources (air sac system respiratory muscles, syrinx) influence online (as song is performed) and offline neural processes in song control circuits. It will also be important to study additional species, including non-vocal learners, to elucidate whether increasing vocal skill over evolutionary time led to more sophistication of the feedback mechanisms.

## 2. Conclusion and outlook

Respiratory activity drives vocalization and, especially during song, exhibits remarkable tempo as well as ability to sufficiently pressurize the system in short timeframes. Over the past 30 years, we have made substantial progress towards understanding the important role of respiration in avian phonation. Respiratory mechanisms give rise to highly remarkable song tempos. This activity needs to be integrated into the vital functions that respiration serves. These vital functions have exerted selective forces on respiratory mechanics and gas exchange. Song as a sexually selected behaviour may put demands on respiration that may experience limitations dictated by non-vocal physiological mechanisms (e.g. acid/base homeostasis and hyperventilation), which are the result of natural selective forces. The insights gained also pose new questions, and in particular the neural integration of respiratory song motor patterns is still incompletely understood, including how peripheral feedback from respiratory muscles and structures contribute to song production and maintenance of stereotyped patterns. The highly dynamic respiratory activity during song also presents a useful behaviour for studying airflow patterns within the air sac–lung system of birds—and can therefore contribute to a better understanding of general control mechanisms of lung ventilation. To address evolutionary questions regarding birdsong, we need physiological research on non-vocal learners, especially basal and more derived taxa within major lineages [78]. Although the emphasis on oscines has yielded highly interesting details, questions concerning the evolution of vocal ability in birds requires a broader taxonomic investigation.

## Data Availability

This article has no additional data.
